# Artificial Intelligence-Assisted Prediction of Immunotherapy Benefit and Recurrence Risk Stratification in Gastric Cancer: A Single-Center Real-World Study

**DOI:** 10.3390/cancers18162582

**Published:** 2026-08-11

**Authors:** Dou-Dou Li, Jie-Yun Zhang, Yi-Yang Zhang, Yu-Feng Yang, Jun-Xi Chen, Xi-Yuan Chen, Xi Chen, Zhi-Huang Hu, Hai-Xia Wu, Chen-Chen Wang

**Affiliations:** 1 School of Clinical Medicine, Shanghai University of Medicine & Health Sciences, Shanghai 201318, China; 17111230019@fudan.edu.cn (D.-D.L.); steven_zhanggg@163.com (Y.-Y.Z.); 17302118534@163.com (Y.-F.Y.); junxilong@163.com (J.-X.C.); 17717053579@163.com (X.-Y.C.); 2 Department of Medical Oncology, Fudan University Shanghai Cancer Center, Department of Oncology, Shanghai Medical College, Fudan University, Shanghai 200032, China; zjy000999@163.com (J.-Y.Z.); zhihuanghu@hotmail.com (Z.-H.H.); 3 Shanghai Jiai Intelligence Co., Ltd., 3-173, 328 Tiantong Rd., Hongkou, Shanghai 200086, China; ac@kdh.ai; 4 Department of Medical Oncology, Fudan University Shanghai Cancer Center, Minhang District, Shanghai 201102, China

**Keywords:** gastric cancer, PD-1 inhibitor, dynamic prediction, landmark analysis, immune remodeling

## Abstract

Immune checkpoint inhibitors targeting the PD-1 pathway have improved outcomes in advanced gastric cancer; however, substantial heterogeneity in treatment response remains. Current prediction strategies mainly rely on pretreatment biomarkers, which provide static assessments and may not capture the evolving interactions between tumor progression and host immunity. This study aimed to develop a dynamic prediction framework integrating longitudinal clinical trajectories and immune remodeling for continuous risk assessment during PD-1 inhibitor therapy. **Methods:** This retrospective study included 171 patients with gastric cancer receiving PD-1 inhibitor-based treatment. A landmark analysis framework was established using sequential 28-day follow-up windows to predict subsequent progressive disease (PD) or recurrence based on available clinical and immune information. Three nested models were developed: a baseline model (M0), a dynamic clinical model (M1), and an immune-integrated model (M2) incorporating longitudinal lymphocyte subset features. Model performance was evaluated using discrimination, calibration, and internal validation with patient-level resampling strategies. **Results:** Predictive performance improved progressively with incorporation of longitudinal information. The ROC AUC increased from 0.559 (95% CI, 0.429–0.690) in M0 to 0.737 (95% CI, 0.613–0.852) in M1 and 0.786 (95% CI, 0.681–0.883) in M2. Dynamic clinical information significantly improved discrimination compared with baseline prediction (ΔAUC = 0.178, *p* = 0.025), while additional immune features further enhanced risk stratification (ΔAUC = 0.049, *p* = 0.040). Interpretation analysis identified tumor burden evolution, neutrophil-to-lymphocyte ratio trajectories, and immune functional axes involving T-cell, innate cytotoxicity, and humoral immunity as major contributors to risk estimation. Landmark analyses demonstrated the feasibility of continuously updating PD/recurrence risk during treatment. **Conclusions:** This study establishes a dynamic landmark prediction framework integrating longitudinal clinical trajectories and immune remodeling for continuous risk assessment during PD-1 inhibitor therapy. Dynamic clinical and immune features provided complementary predictive information beyond baseline characteristics, supporting an adaptive approach for precision immunotherapy monitoring in gastric cancer.

## Simple Summary

Immune checkpoint inhibitors targeting PD-1 have improved outcomes for patients with advanced gastric cancer, but only a subset of patients achieve durable benefit. Current prediction approaches mainly rely on baseline biomarkers and may not reflect changes occurring during treatment. In this study, we developed a dynamic prediction framework that continuously evaluates disease progression or recurrence risk during PD-1 inhibitor therapy by integrating changes in clinical characteristics and immune profiles over time. Using data from 171 patients, we found that treatment-related clinical changes and immune remodeling provided additional predictive information beyond baseline features. This approach enables repeated risk assessment throughout treatment and may support more personalized monitoring and management of patients receiving immunotherapy.

## 1. Introduction

Gastric cancer remains one of the most common malignancies of the digestive system and a leading cause of cancer-related mortality worldwide, ranking among the top five cancers in both incidence and mortality [1,2]. The disease burden is particularly pronounced in East Asia, especially in China, which accounts for more than one-third of global new cases and nearly 40% of gastric cancer-related deaths [3]. Due to the lack of specific symptoms at the early stage, most patients are diagnosed at locally advanced or metastatic stages, resulting in poor prognosis [4]. Despite significant advances in multimodal therapies, including surgery, chemotherapy, radiotherapy, targeted therapy, and immunotherapy, the outcomes of advanced gastric cancer remain unsatisfactory [5,6]. This considerable heterogeneity highlights the need for more precise approaches to characterize individual treatment trajectories and optimize immunotherapy management.

In recent years, immune checkpoint inhibitors targeting the PD-1/PD-L1 axis have markedly transformed the therapeutic landscape of advanced gastric cancer and become an essential component of systemic treatment [7,8,9]. By restoring antitumor immune responses, PD-1 blockade has provided durable clinical benefits in a subset of patients, as demonstrated by multiple phase III trials, including CheckMate 649, KEYNOTE-859, and ATTRACTION-2 [10,11,12]. However, substantial heterogeneity remains among treated patients, with durable responses more frequently observed in individuals with favorable immune-related characteristics, such as high PD-L1 expression, microsatellite instability-high/deficient mismatch repair (MSI-H/dMMR), Epstein–Barr virus-positive tumors, and elevated tumor mutational burden. Current response prediction mainly relies on these pretreatment biomarkers and clinicopathological features, which provide valuable information regarding baseline tumor biology and immune context [13]. Nevertheless, these indicators represent a static snapshot before treatment initiation and may not fully capture the dynamic interactions between tumor evolution and host immunity during immunotherapy [14].

The time-dependent nature of immunotherapy response suggests that longitudinal assessment strategies may provide complementary information beyond conventional baseline prediction [15,16]. Despite advances in biomarker discovery and immune monitoring, substantial heterogeneity remains in treatment trajectories among patients receiving PD-1 blockade, and reliable approaches for continuously assessing individual risk are still lacking [17]. Recent advances in immune profiling and real-world clinical data have enabled the characterization of treatment-associated changes in circulating immune populations, inflammatory status, and clinical trajectories [18,19]. Nevertheless, translating these dynamic measurements into clinically applicable prediction frameworks remains challenging [20]. Existing prediction models are predominantly designed for baseline risk classification and often fail to incorporate sequential information collected during treatment. Moreover, longitudinal immune features are frequently high-dimensional and heterogeneous, requiring approaches that can integrate multiple time-dependent variables while maintaining interpretability and minimizing potential information leakage during model development [21]. Therefore, a clinically applicable framework capable of continuously updating individual risk according to evolving clinical and immune profiles remains an unmet need in gastric cancer immunotherapy.

In this study, we developed and internally validated a dynamic landmark prediction framework to continuously assess PD/recurrence risk during PD-1 inhibitor therapy in patients with gastric cancer. Unlike conventional models based on pretreatment characteristics, our framework incorporated longitudinal clinical trajectories and dynamic lymphocyte subset alterations collected during treatment, enabling repeated risk updating at sequential time points. Three nested models were constructed to quantify the incremental contribution of dynamic clinical information and immune remodeling beyond baseline characteristics. Through patient-level validation and comprehensive evaluation of discrimination, calibration, and clinical utility, we investigated whether longitudinal clinical and immune features could improve individualized risk stratification during immunotherapy. This study provides a clinically interpretable approach for transforming static baseline prediction into adaptive monitoring of treatment trajectories and may facilitate more precise surveillance and management of gastric cancer patients receiving PD-1 inhibitor therapy.

## 2. Materials and Methods

### 2.1. Patients

This multicenter cohort study included patients with histologically confirmed gastric cancer who received PD-1 inhibitor-based therapy and had available clinical, laboratory, imaging, and immune monitoring data. The study incorporated retrospective and prospective data collection to develop and internally validate a dynamic prediction model for treatment outcomes in patients undergoing immunotherapy. Eligible patients were required to have complete baseline clinicopathological information, documented PD-1 inhibitor treatment exposure, and evaluable clinical outcomes during follow-up. The date of the first PD-1 inhibitor administration was defined as day 1 (D1), and longitudinal clinical, laboratory, imaging, and immune-related data were collected from treatment initiation throughout follow-up. Patients with missing key identifiers that prevented data linkage, duplicate records, unavailable outcome assessment, or unresolved chronological inconsistencies were excluded from the analysis. The study was conducted in accordance with the Transparent Reporting of a multivariable prediction model for Individual Prognosis or Diagnosis (TRIPOD) guidelines for prediction model development and validation. The study protocol was approved by the institutional ethics committees of participating centers and was conducted in accordance with the Declaration of Helsinki.

### 2.2. Study Design and Treatment

Patients received PD-1 inhibitor-based immunotherapy according to routine clinical practice, including PD-1 inhibitor monotherapy or combination regimens with chemotherapy or other systemic treatments. The date of the first PD-1 inhibitor administration was defined as day 1 (D1). Tumor response was evaluated according to the Response Evaluation Criteria in Solid Tumors (RECIST) version 1.1 based on radiological assessments, including computed tomography (CT) or magnetic resonance imaging (MRI), performed at baseline and during follow-up. Clinical outcomes, including progressive disease (PD) and recurrence events, were systematically collected throughout the observation period.

The present study was developed based on the data structure of a 171-patient gastric cancer cohort receiving PD-1 inhibitor therapy. The analytical framework integrated baseline clinicopathological characteristics, longitudinal 28-day follow-up information, dynamic lymphocyte subset monitoring, and PD/recurrence outcomes. To characterize treatment response dynamically and minimize guarantee-time bias, a landmark analysis framework was applied. Follow-up after PD-1 initiation was divided into eight consecutive 28-day windows: W1 (D1–28), W2 (D29–56), and sequentially through W8 (D197–224). At the end of each landmark window Wk, patients who remained free of PD or recurrence were included in the corresponding landmark risk set. Information available up to the end of Wk was used to predict the first occurrence of PD or recurrence during the subsequent window W(k + 1). Patients who experienced PD or recurrence before the landmark time point were excluded from the corresponding risk set. To minimize potential information leakage, all longitudinal observations from the same patient were assigned to the same data partition during model validation, ensuring that different time windows from an individual patient were not simultaneously included in training and validation datasets.

### 2.3. Variable System

Study variables were predefined according to the hierarchical structure of the prediction models and included baseline clinicopathological variables, longitudinal clinical dynamic variables, and longitudinal immune-related variables. Three nested models were constructed to evaluate the incremental predictive value of dynamic clinical information and immune monitoring features. The baseline model (M0) included pretreatment characteristics collected before PD-1 inhibitor initiation, including age, sex, TNM stage, pathological subtype, tumor size, previous treatment history, and other baseline clinical variables. The clinical dynamic model (M1) incorporated all variables from M0 together with longitudinal clinical information available up to each landmark time point, including treatment exposure, treatment-related characteristics, symptoms, routine laboratory examinations, and imaging follow-up data. The immune-integrated model (M2) further incorporated longitudinal lymphocyte subset features based on M1. Immune-related variables were derived from flow cytometry-based immune monitoring and included current values, relative changes from baseline, adjacent-window changes, and temporal slopes of immune parameters during PD-1 inhibitor therapy. The evaluated immune features included total T lymphocytes (CD3^+^), helper T lymphocytes (CD4^+^), cytotoxic T lymphocytes (CD8^+^), CD4/CD8 ratio, natural killer (NK) cells, and CD19^+^ B cells, which were used to characterize dynamic immune changes and assess their additional contribution beyond baseline and clinical dynamic variables.

### 2.4. Data Processing and Dynamic Feature Extraction

Structured data cleaning and harmonization were performed before model development. Within the landmark framework, all dynamic features were calculated using only information available before or at the end of each landmark window Wk to preserve temporal ordering and avoid information leakage. Longitudinal clinical and immune features were derived from repeated measurements during PD-1 inhibitor therapy, including current values, relative changes from baseline, adjacent-window changes, and temporal slopes. Dynamic immune features were constructed from flow cytometry-based lymphocyte subset monitoring, and variables were included only when predefined data availability criteria were met. Missing values were imputed and continuous variables were standardized using parameters derived exclusively from the training folds during cross-validation. Immune-related variables were further summarized into functional immune axes to improve biological interpretation. All feature engineering procedures were performed within the training data only to prevent information leakage between model development and validation datasets.

### 2.5. Model Development

Three predefined nested models were developed to evaluate the incremental predictive value of longitudinal clinical and immune features. The baseline model (M0) incorporated pretreatment clinicopathological variables, the clinical dynamic model (M1) further included longitudinal clinical information available up to each landmark window, and the immune-integrated model (M2) additionally incorporated dynamic lymphocyte subset features. All models were developed using regularized logistic regression to provide interpretable risk estimates and reduce overfitting in the relatively limited cohort. Internal validation was performed using repeated five-fold patient-grouped cross-validation, in which all longitudinal observations from the same patient were assigned to the same fold to avoid information leakage. Out-of-fold predicted probabilities were generated for model evaluation, and all preprocessing procedures, including missing-value handling, feature standardization, and model recalibration, were performed exclusively within the training folds. The incremental predictive value of additional feature sets was assessed by comparing M1 with M0 and M2 with M1, and model performance was evaluated based on discrimination, calibration, and overall predictive accuracy metrics.

### 2.6. Cohort Stratification and Model Validation

Patients were stratified according to predefined analytical objectives. For the final treatment benefit analysis, the outcome was defined as clinical benefit (partial response [PR] or stable disease [SD]) versus non-benefit, and all eligible patients with evaluable treatment response were included. For dynamic landmark analyses, the landmark risk set at each window Wk consisted of patients who remained free of PD or recurrence at the end of that window and had available follow-up information in the subsequent window W(k + 1). Repeated patient-level cross-validation was used for internal validation, ensuring that all observations from the same patient were consistently assigned to the same data partition. Model performance was evaluated using discrimination, calibration, and predictive accuracy metrics, including ROC AUC, PR AUC, Brier score, calibration intercept, calibration slope, integrated calibration index (ICI), and expected calibration error (ECE). Confidence intervals were estimated using patient-level bootstrap resampling, and window-specific landmark analyses were evaluated using patient-cluster bootstrap to account for repeated observations within individuals. The incremental performance of nested models was assessed by comparing paired predictions between M0, M1, and M2 using appropriate statistical tests.

### 2.7. Statistical Analysis

The cleaning and integration of raw clinical data were performed using the OncologyAI platform (version 1.3) developed by KDH.ai. All statistical analyses were conducted using R software (version 4.2.1) and RStudio (version 2022.07.1). Data processing and model training were performed in Python (version 3.8.5) with the TensorFlow framework (version 2.4.0). Continuous variables were summarized using appropriate descriptive statistics, and categorical variables were presented as frequencies and percentages. Model performance comparisons were conducted using paired DeLong tests for differences in ROC AUC and patient-level bootstrap resampling for other performance metrics. Confidence intervals were estimated using bootstrap methods, with patient-cluster bootstrap applied for landmark analyses involving repeated patient-window observations. Calibration performance was assessed using calibration intercept, calibration slope, Brier score, integrated calibration index (ICI), and expected calibration error (ECE). All statistical tests were two-sided, and a *p* value < 0.05 was considered statistically significant.

## 3. Results

### 3.1. Baseline Characteristics

A total of 171 patients with gastric cancer receiving PD-1 inhibitor-based therapy were included in the final analysis cohort. According to treatment response, patients were categorized into a benefit group (partial response [PR] or stable disease [SD], *n* = 47) and a non-benefit group (progressive disease [PD], *n* = 124). The baseline demographic, clinicopathological, and laboratory characteristics of the two groups are summarized in Table 1. The median age was comparable between the benefit and non-benefit groups (62.0 vs. 60.0 years), and male patients accounted for the majority of both groups (68.1% vs. 66.9%). The cohort exhibited heterogeneous disease characteristics, including different pathological stages and histological subtypes, with adenocarcinoma and poorly differentiated adenocarcinoma representing the predominant pathological categories. Baseline laboratory parameters, including albumin, C-reactive protein, hemoglobin, and neutrophil-to-lymphocyte ratio, as well as tumor size and previous surgical history, showed no significant differences between groups. In contrast, disease stage and ECOG performance status differed significantly, with patients without treatment benefit exhibiting more advanced disease and poorer baseline functional status. These findings indicate that conventional pretreatment characteristics captured part of the clinical heterogeneity associated with PD-1 inhibitor outcomes but were insufficient to fully characterize treatment trajectories, supporting the integration of longitudinal clinical and immune dynamic features in subsequent analyses.

### 3.2. Patient Flowchart and Dynamic Landmark Cohort

**Figure 1:**
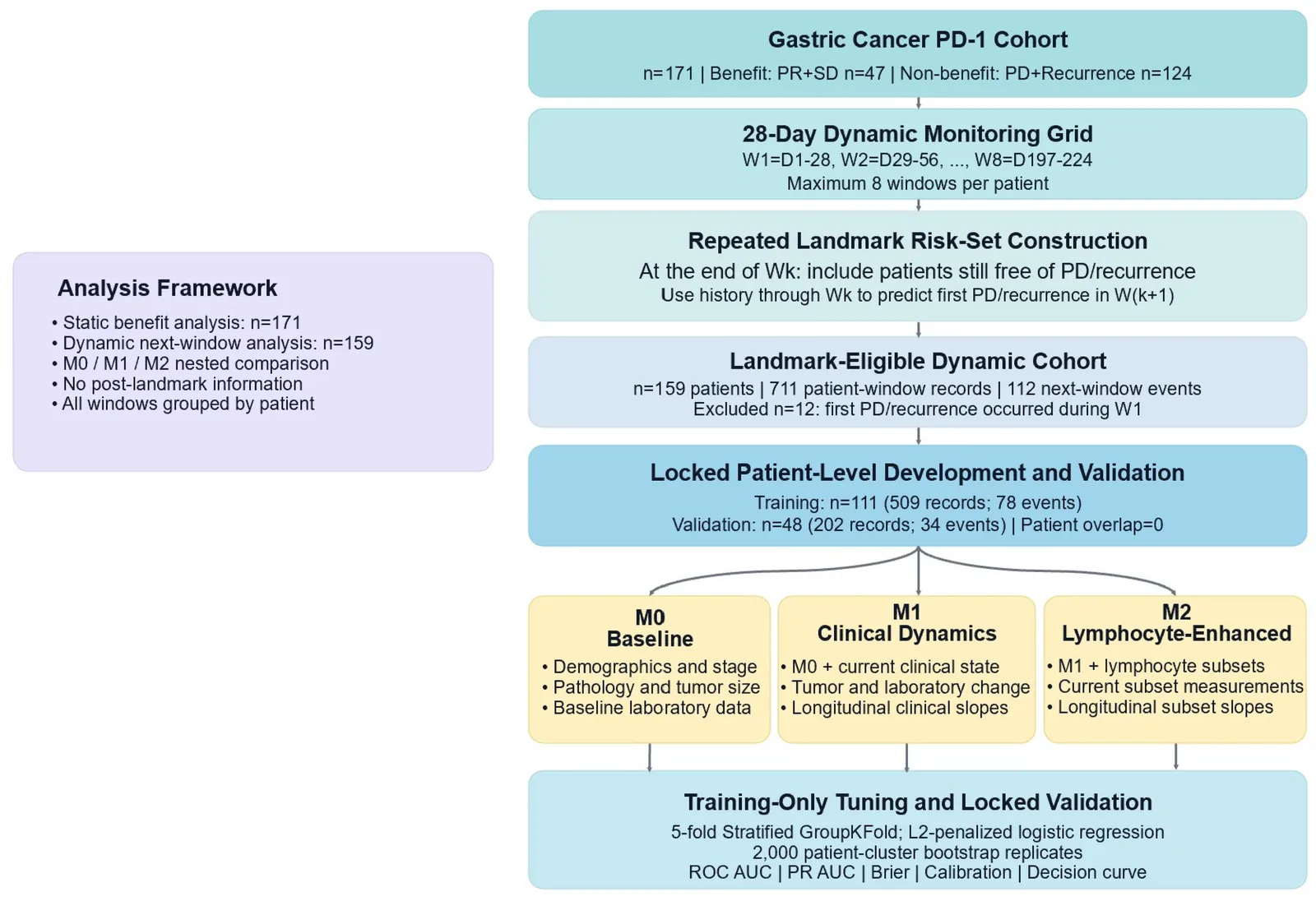
**Dynamic landmark framework for longitudinal prediction of immunotherapy outcomes in gastric cancer.** A total of 171 patients receiving PD-1 inhibitor therapy were included. Longitudinal clinical, laboratory, lymphocyte-subset, and outcome data were organized into 28-day landmark windows. At each landmark, patients remaining at risk were used to construct risk sets for predicting the first PD/recurrence event in the subsequent window. Three nested models were developed: M0, a baseline model incorporating pretreatment characteristics; M1, a dynamic clinical model incorporating longitudinal clinical trajectories; and M2, an immune-enhanced model additionally incorporating lymphocyte-subset dynamics. Patient-level data splitting was applied throughout model development and validation to avoid information leakage. Models were evaluated using cross-validation, bootstrap resampling, discrimination, calibration, and decision-curve analyses.

Following PD-1 inhibitor initiation, a longitudinal landmark cohort was established to characterize treatment trajectories and enable dynamic prediction of subsequent disease progression. The overall study workflow, including patient inclusion, longitudinal follow-up, landmark window definition, dynamic feature extraction, and model development, is illustrated in Figure 1. The final cohort consisted of 171 patients with gastric cancer receiving PD-1 inhibitor-based therapy, with the first administration date defined as day 1 (D1). Follow-up was divided into eight consecutive 28-day windows (W1: D1–28, W2: D29–56, and sequentially through W8: D197–224). At the end of each landmark window Wk, patients who remained free of progressive disease (PD) or recurrence were included in the corresponding risk set, and all clinical and immune information available up to that time point was used to predict the first PD or recurrence event during the subsequent window W(k + 1).

During longitudinal follow-up, 159 patients (93.0% of the initial cohort) entered at least one subsequent landmark risk set, generating 711 patient-window records across W1–W7. A total of 112 first PD or recurrence events occurred during subsequent windows, corresponding to an event rate of 15.8% at the patient-window level. Baseline characteristics according to recurrence status were additionally evaluated to describe clinical heterogeneity associated with subsequent disease events (Table 2). The composition of each landmark window, including the number of patients entering each risk set, follow-up availability, lymphocyte subset monitoring coverage, and event distribution, is summarized in Table 3. The number of eligible patients and observed events gradually decreased in later windows; therefore, W8 was not evaluated because no subsequent W9 outcome was available. In particular, the W7 analysis included only 49 patients with two subsequent events and was considered exploratory because of the limited number of outcome events.

### 3.3. Performance of Nested Prediction Models

**Figure 2:**
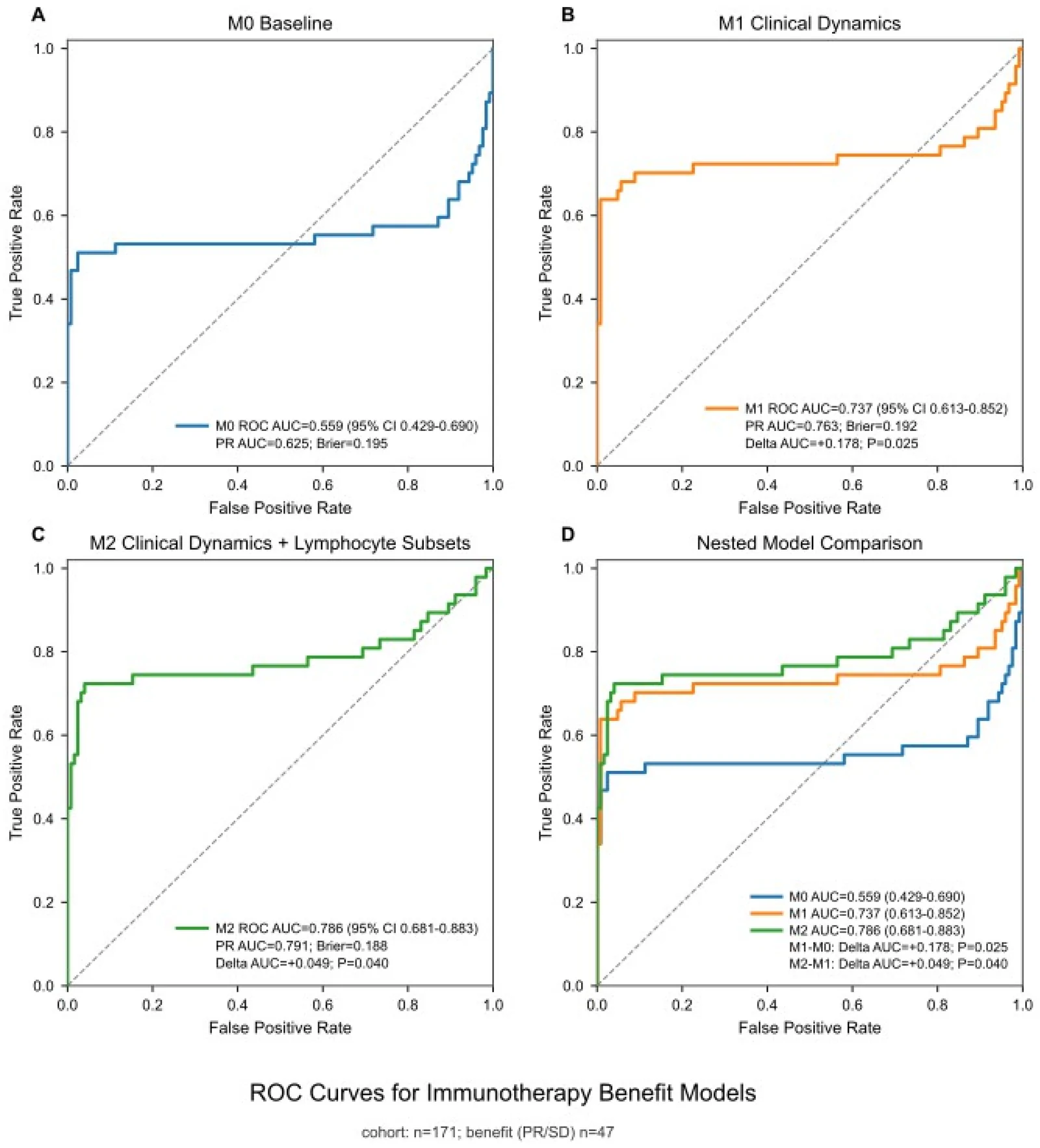
Receiver operating characteristic (ROC) curves of nested immunotherapy-benefit prediction models in the cohort (*n* = 171; benefit defined as PR/SD, *n* = 47). (**A**) ROC curve of the baseline model (M0), showing an AUC of 0.559 (bootstrap 95% CI, 0.429–0.690). (**B**) ROC curve of the clinical dynamics model (M1), demonstrating improved discrimination compared with M0 (AUC = 0.737; 95% CI, 0.613–0.852; ΔAUC = 0.178; DeLong test, *p* = 0.025). (**C**) ROC curve of the combined model (M2) incorporating clinical dynamics and lymphocyte-subset information, showing further improvement (AUC = 0.786; 95% CI, 0.681–0.883; ΔAUC = 0.049; DeLong test, *p* = 0.040). (**D**) Comparison of ROC curves among the three nested models (M0, M1, and M2).

**Figure 3:**
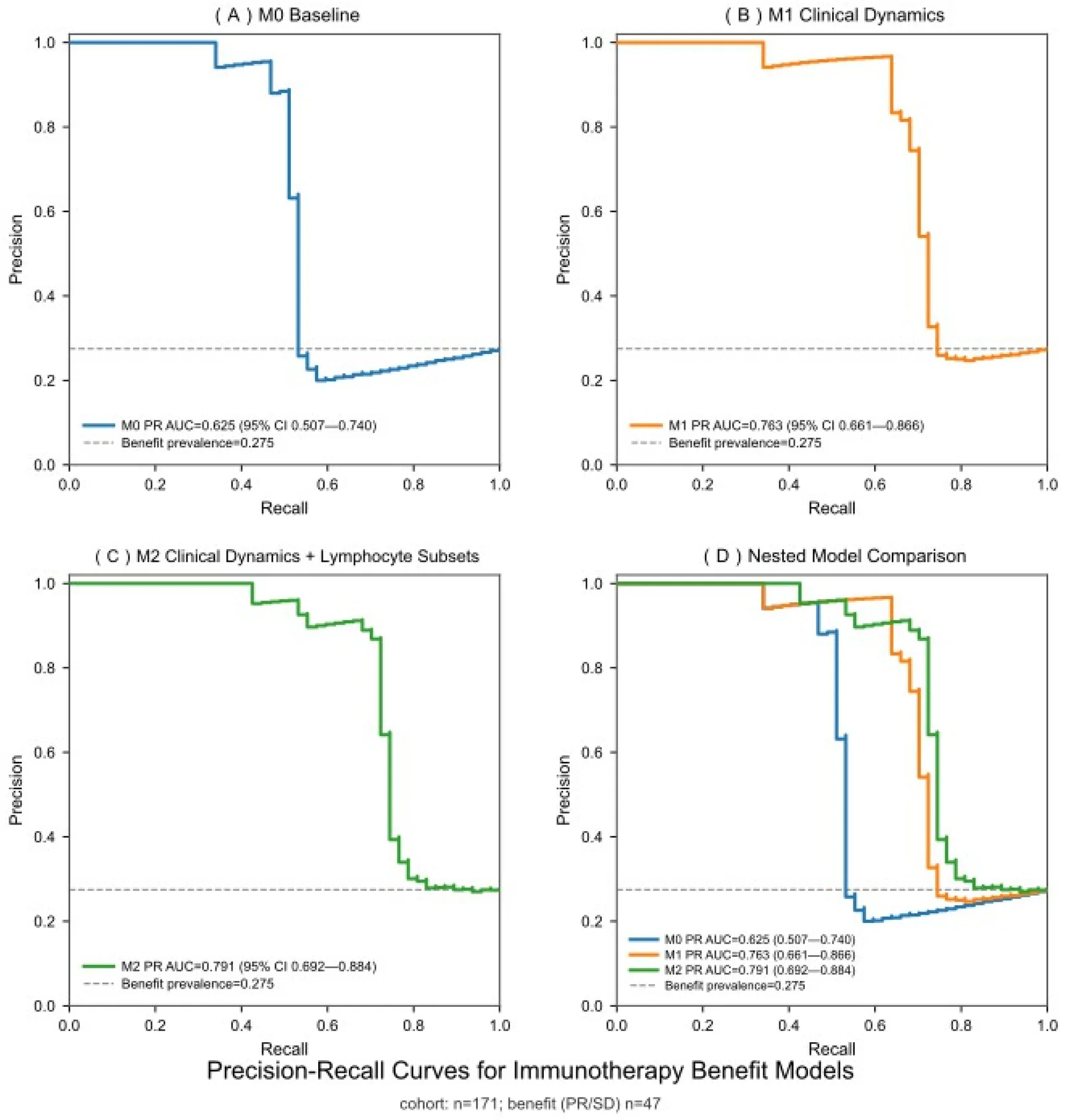
Precision-recall (PR) curves of nested immunotherapy-benefit prediction models in the cohort (*n* = 171; benefit defined as PR/SD, *n* = 47; prevalence, 0.275). (**A**) PR curve of the baseline model (M0), showing a PR AUC of 0.625 (bootstrap 95% CI, 0.507–0.740). (**B**) PR curve of the clinical dynamics model (M1), demonstrating improved performance with a PR AUC of 0.763 (95% CI, 0.661–0.866). (**C**) PR curve of the combined model (M2) incorporating clinical dynamics and lymphocyte-subset information, showing further improvement (PR AUC = 0.791; 95% CI, 0.692–0.884). (**D**) Comparison of PR curves among the three nested models (M0, M1, and M2). The horizontal dashed line indicates the prevalence of immunotherapy benefit in the cohort.

Three predefined nested models were developed to evaluate the incremental predictive value of longitudinal clinical and immune features for PD-1 inhibitor outcomes. The baseline model (M0) incorporated pretreatment clinicopathological characteristics, the clinical dynamic model (M1) further included longitudinal clinical information available up to each landmark time point, and the immune-integrated model (M2) additionally incorporated dynamic lymphocyte subset features. Progressive improvement in model discrimination was observed with sequential integration of dynamic information. ROC analysis demonstrated that M1 and M2 achieved higher discrimination than M0, with further improvement after incorporating lymphocyte subset dynamics (Figure 2 and Figure 3). Specifically, the ROC AUC increased from 0.559 (95% CI, 0.429–0.690) in M0 to 0.737 (95% CI, 0.613–0.852) in M1 and further to 0.786 (95% CI, 0.681–0.883) in M2, indicating that longitudinal clinical trajectories and immune remodeling provided additional information beyond baseline characteristics.

**Figure 4:**
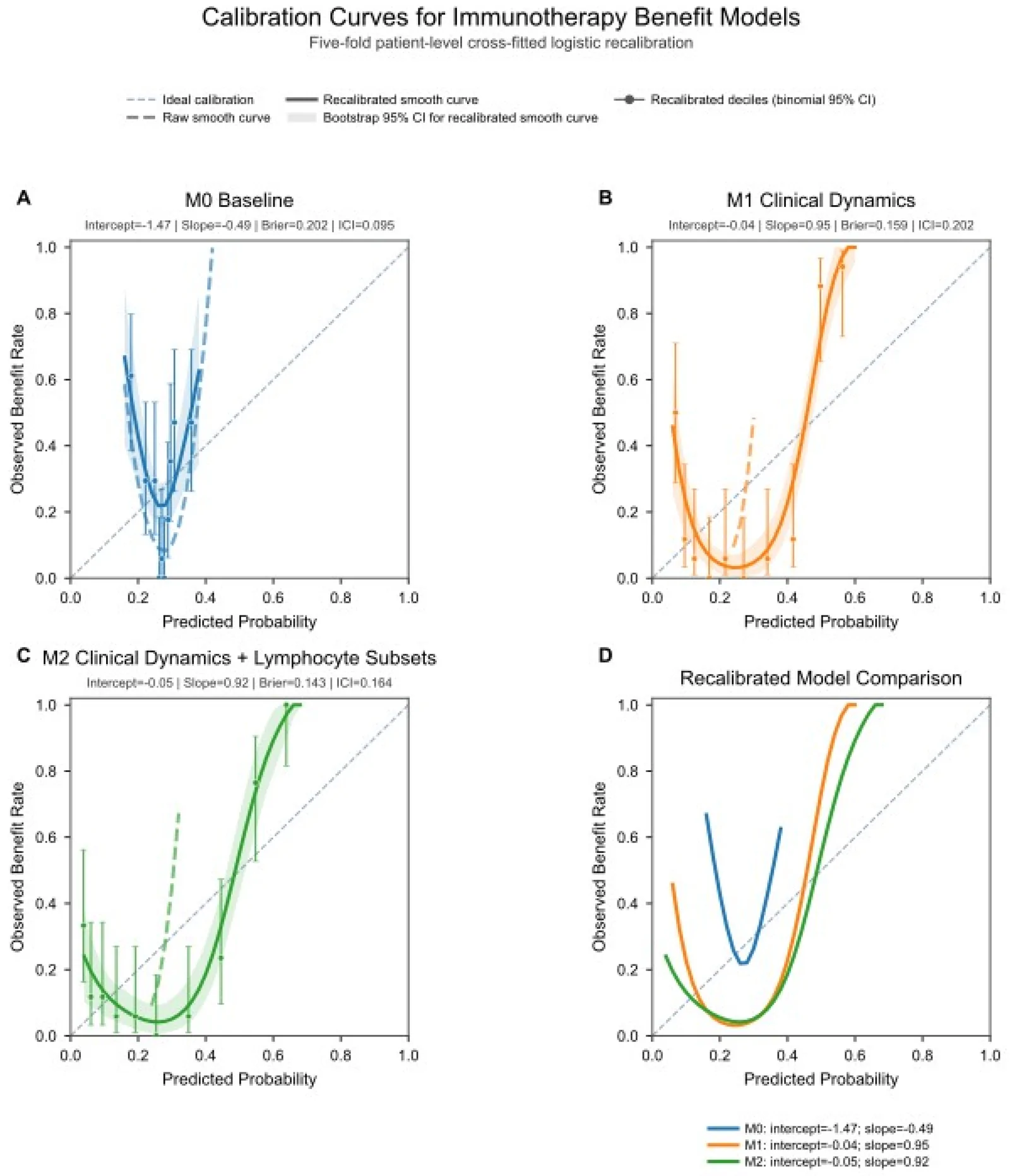
**Internal calibration of nested models incorporating dynamic clinical and lymphocyte-subset features for immunotherapy benefit prediction.** Internal calibration performance of nested immunotherapy-benefit prediction models after patient-level cross-fitted logistic recalibration. Calibration curves for M0 (baseline model), M1 (incorporating dynamic clinical features), and M2 (further incorporating lymphocyte-subset dynamics) are shown in panels (**A**–**C**). Dashed curves represent calibration before recalibration, whereas solid curves represent calibration after five-fold patient-level cross-fitted logistic recalibration. Shaded areas indicate bootstrap 95% confidence intervals, and points represent recalibrated probability deciles with corresponding binomial 95% confidence intervals. Panel (**D**) compares the recalibrated calibration curves across the three models. The diagonal line indicates ideal calibration. Cross-fitted calibration intercepts/slopes were −1.47/−0.49 for M0, −0.04/0.95 for M1, and −0.05/0.92 for M2, respectively. Corresponding Brier scores were 0.202, 0.159, and 0.143, and integrated calibration indices were 0.095, 0.202, and 0.164 for M0, M1, and M2, respectively.

Consistent with ROC analysis, PR analysis showed improved performance after incorporating dynamic features, particularly in the context of an imbalanced clinical outcome distribution (Figure 4). The PR AUC increased from 0.625 (95% CI, 0.507–0.740) for M0 to 0.763 (95% CI, 0.661–0.866) for M1 and 0.791 (95% CI, 0.692–0.884) for M2. Detailed model comparisons demonstrated that M1 significantly improved ROC AUC by 0.178 (95% CI, 0.025–0.326; *p* = 0.025) and PR AUC by 0.138 (95% CI, 0.005–0.270; *p* = 0.046) compared with M0. Compared with M1, M2 provided an additional improvement in ROC AUC of 0.049 (95% CI, 0.008–0.101; *p* = 0.040), whereas the improvement in PR AUC did not reach statistical significance (Table 4). These findings suggest that longitudinal clinical information accounted for the major gain in predictive discrimination, while dynamic lymphocyte subset features further enhanced model refinement.

### 3.4. Model Calibration and Validation

**Figure 5:**
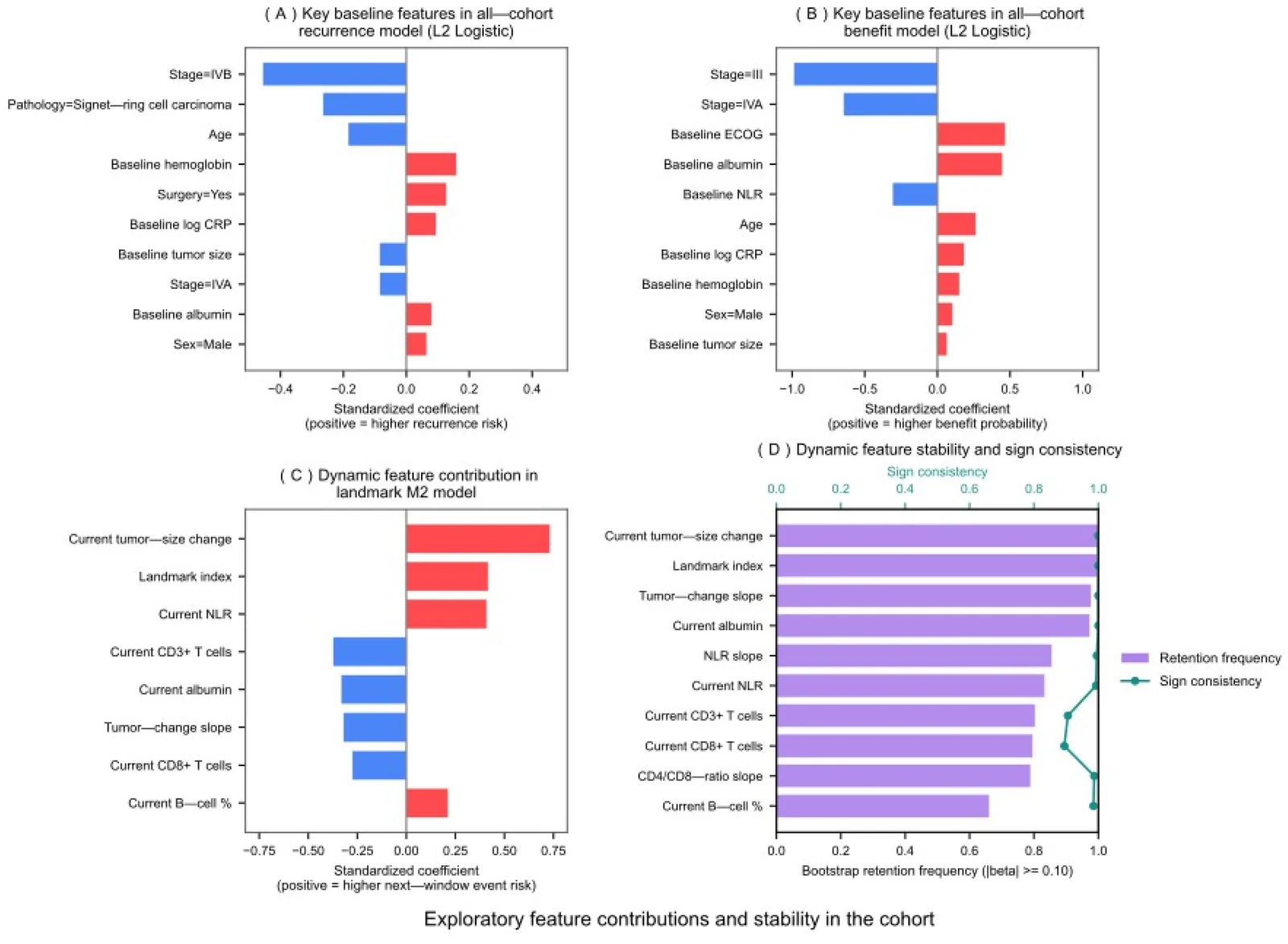
**Exploratory feature contributions and stability analysis of clinical and immune-related predictors in the cohort.** (**A**) Baseline features with the largest absolute standardized coefficients from an all-cohort L2-penalized logistic regression model for recurrence prediction (48 recurrence events among 171 patients). (**B**) Baseline features with the largest absolute standardized coefficients from an all-cohort L2-penalized logistic regression model for immunotherapy-benefit prediction (benefit defined as PR/SD; *n* = 47). (**C**) Standardized coefficients of the leading dynamic features from the pooled landmark M2 model for next-window progression or recurrence prediction. Red and blue bars indicate positive and negative coefficients, respectively. (**D**) Patient-cluster bootstrap stability analysis of the leading dynamic features using a reduced ridge-logistic landmark model (500 bootstrap replicates). Purple bars represent the proportion of bootstrap models retaining features with an absolute coefficient magnitude (|β|) ≥ 0.10, and the teal line indicates coefficient sign consistency among retained models. Panels (**A**,**B**) represent exploratory refits and were not used to derive the prespecified synthetic prediction scores.

**Figure 6:**
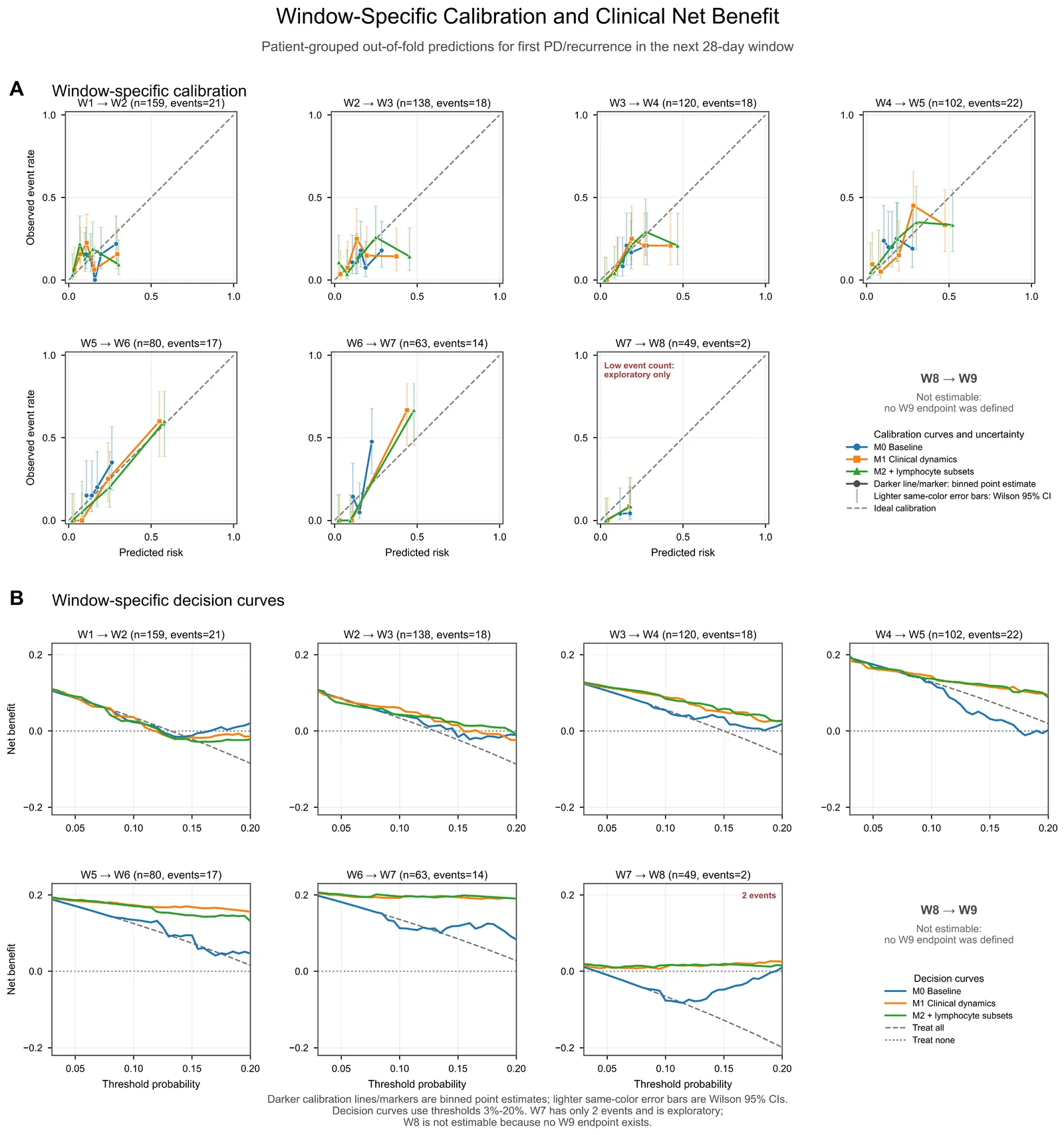
**Window-specific calibration and clinical net benefit of dynamic landmark models for predicting next-window progression or recurrence.** (**A**) Calibration curves for M0 (baseline), M1 (baseline plus dynamic clinical features), and M2 (M1 plus lymphocyte-subset dynamics) across sequential 28-day landmark windows. Darker lines indicate binned estimates, and lighter error bars indicate 95% confidence intervals from 500 patient-cluster bootstrap samples. (**B**) Decision curves for M0, M1, and M2 across threshold probabilities. Dashed and dotted gray lines indicate the treat-all and treat-none strategies, respectively. W8 was not estimable because no subsequent prediction window was defined.

Beyond discrimination performance, the calibration and robustness of the nested prediction models were further evaluated. Calibration analysis demonstrated improved agreement between predicted and observed outcomes after incorporating longitudinal clinical and immune features (Figure 5). Compared with the baseline model M0, the dynamic models M1 and M2 showed closer calibration curves, with calibration intercepts and slopes approaching the ideal values and lower Brier scores, indicating more reliable individual risk estimation. Internal validation using repeated patient-level cross-validation and patient-cluster bootstrap resampling further confirmed the stability of model performance (Figure 6). The validated performance distributions showed consistent improvement of M1 and M2 over M0, with reduced prediction uncertainty and limited evidence of overfitting. Moreover, decision-curve analysis demonstrated higher net benefit of the dynamic models across clinically relevant threshold probabilities. Together, these findings indicate that incorporating longitudinal clinical trajectories and lymphocyte subset dynamics improved not only predictive discrimination but also calibration stability and potential clinical utility for individualized prediction of PD/recurrence during PD-1 inhibitor therapy.

### 3.5. Interpretation of Dynamic Features

**Figure 7:**
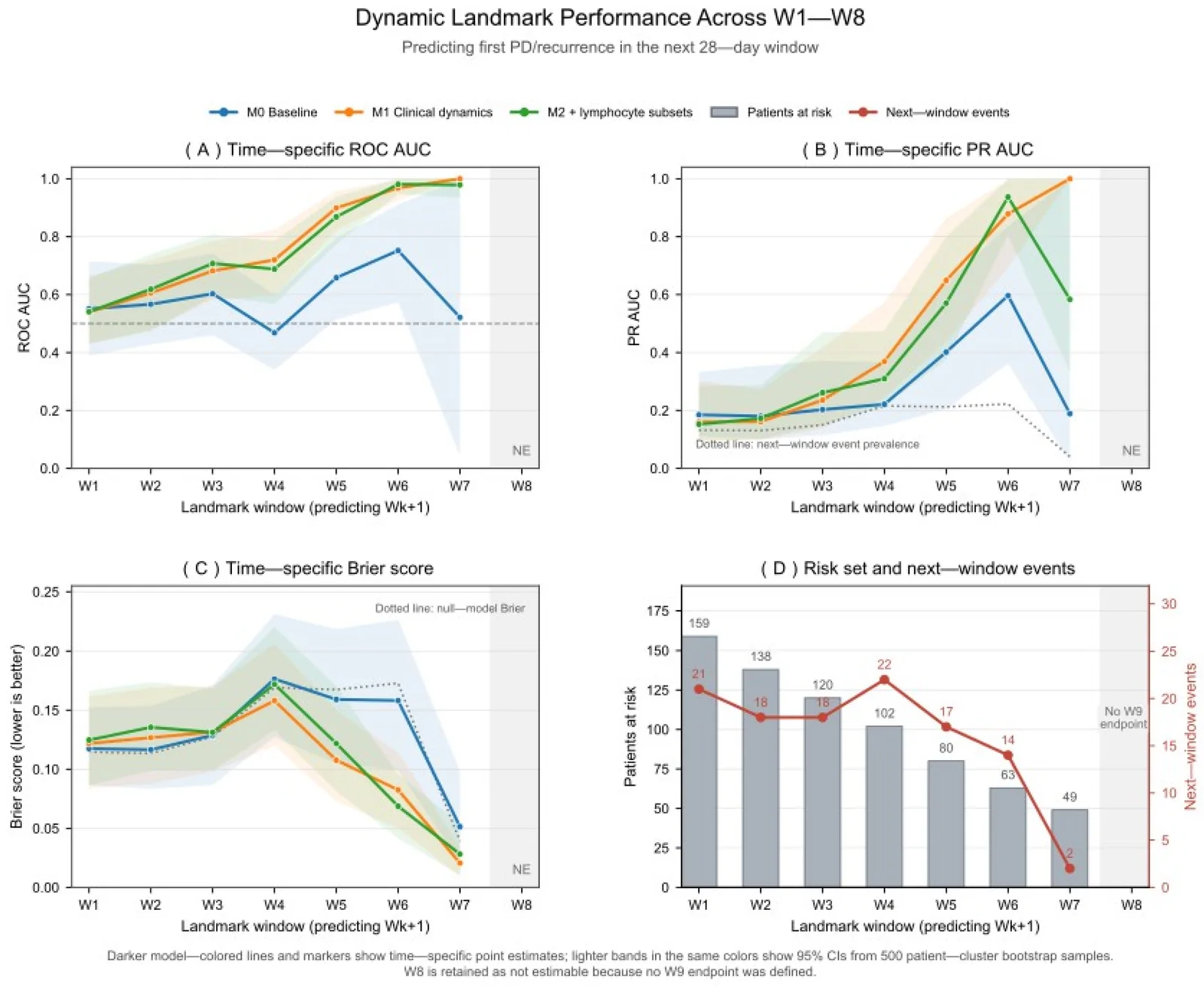
**Event-aligned trajectories reveal dynamic risk escalation and lymphocyte-subset changes preceding progression or recurrence.** Landmark windows were aligned relative to the first progressive disease (PD)/recurrence event, with W−1 representing the landmark window immediately preceding the event window. (**A**) Mean predicted next-window risk trajectories from M0 (baseline model), M1 (baseline plus dynamic clinical features), and M2 (M1 plus lymphocyte-subset dynamics) among patients approaching an event. (**B**) M2-predicted risk trajectories in event patients compared with a weighted benefit reference group matched according to the event landmark distribution. (**C**) Longitudinal trajectories of standardized lymphocyte subsets, including CD3+ T cells, CD8+ T cells, CD4/CD8 ratio, and CD19+ B cells, before PD/recurrence. (**D**) Differences in standardized lymphocyte-subset levels between event patients and the weighted benefit reference group, with negative values indicating lower levels in event patients. Lymphocyte-subset values were standardized using all available lymphocyte-panel measurements. Lines indicate mean trajectories, and shaded regions represent 95% confidence intervals obtained from 500 patient-level bootstrap resampling iterations.

Feature contribution analysis of the integrated M2 model provided insights into the clinical and immune determinants underlying dynamic risk prediction (Figure 7). Among the longitudinal clinical variables, changes in tumor burden represented the most influential predictor of subsequent PD/recurrence risk, indicating that treatment-associated disease evolution provided substantially more information than baseline tumor characteristics alone. Systemic inflammatory indicators, particularly the neutrophil-to-lymphocyte ratio (NLR) and its temporal trajectory, also showed important contributions to risk estimation, suggesting that dynamic inflammatory responses during PD-1 inhibitor therapy were closely associated with subsequent treatment outcomes. In addition to clinical variables, immune-related features displayed heterogeneous contributions to model prediction, highlighting the importance of capturing longitudinal immune alterations rather than relying on single-time-point immune measurements. The directional associations and biological interpretation of these variables were further summarized according to predefined functional immune axes (Table 5). Dynamic features related to lymphocyte immune reserve, total T-cell abundance, helper T-cell function, cytotoxic T-cell activity, immune balance, innate cytotoxicity, and humoral immunity demonstrated distinct associations with future PD/recurrence risk. Collectively, these findings indicate that the predictive value of the M2 model was derived from the integrated contribution of tumor burden evolution, systemic inflammatory status, and immune remodeling during treatment, supporting the biological relevance of incorporating longitudinal clinical and immune features into individualized risk assessment.

### 3.6. Temporal Performance of Landmark Prediction

**Figure 8:**
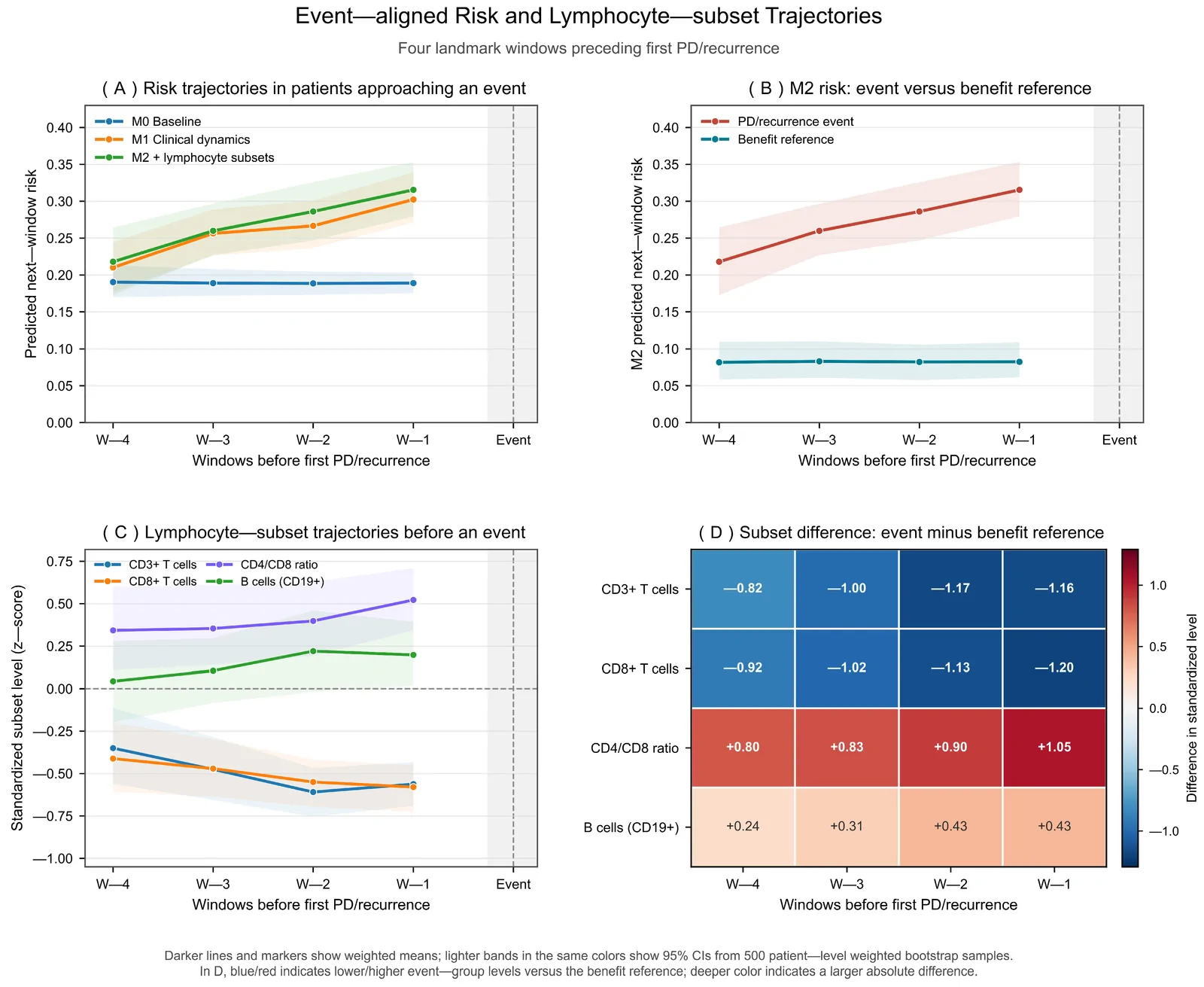
**Lymphocyte-subset coverage and incremental predictive performance of the immune-enhanced landmark model.** (**A**) Patient-window coverage matrix across eight 28-day landmark windows for 171 patients, ordered by treatment benefit status and first PD/recurrence window. Teal cells indicate windows with available follow-up and lymphocyte-panel measurements, red cells indicate the first PD/recurrence window, and gray cells indicate structurally unavailable observations after the event. (**B**) Number of patients at risk with available lymphocyte-panel measurements and structurally unavailable post-event observations across landmark windows. All patients remaining at risk had scheduled lymphocyte-panel measurements in each evaluable window. (**C**) Incremental discrimination of M2 compared with M1 across landmark windows, assessed by differences in ROC AUC and PR AUC; positive values indicate improved performance with M2. (**D**) Incremental prediction error of M2 compared with M1, assessed by differences in Brier score; negative values indicate improved performance with M2. Points represent observed differences, and error bars indicate 95% confidence intervals derived from patient-cluster bootstrap resampling. W8 was excluded from incremental performance analyses because no subsequent prediction window was defined.

To evaluate whether the proposed framework could provide continuous risk updating during PD-1 inhibitor therapy, a series of landmark analyses was performed across sequential 28-day follow-up windows. At the end of each landmark window, patients remaining free of PD or recurrence were included in the corresponding risk set, and available clinical and immune information was used to predict the occurrence of PD or recurrence in the subsequent window. The temporal performance of the landmark models across follow-up windows is summarized in Figure 8. The model maintained predictive capability across multiple early and intermediate follow-up windows, supporting the feasibility of dynamic risk assessment based on evolving clinical and immune information rather than fixed baseline characteristics. However, the number of eligible patients and observed events gradually decreased in later windows, resulting in increased uncertainty of performance estimates. In particular, the W7 prediction included only a limited number of subsequent events and was therefore considered exploratory. These findings indicate that the landmark framework enables longitudinal updating of PD/recurrence risk during PD-1 therapy, while emphasizing the need for larger multicenter cohorts to further validate model stability, particularly in late treatment periods.

### 3.7. Dynamic Risk and Immune Trajectories

**Figure 9:**
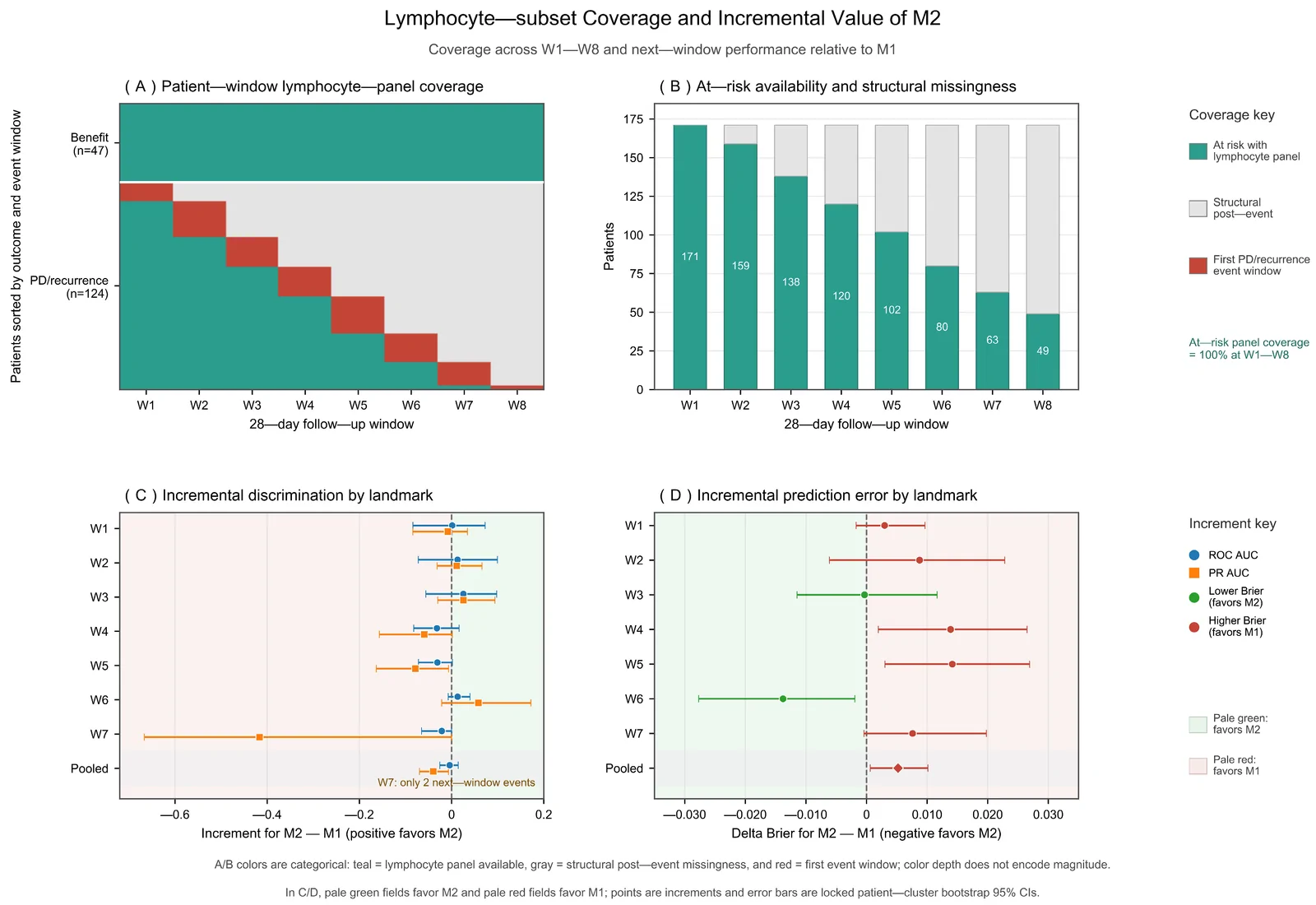
**Internal validation and calibration stability assessment of pooled landmark models.** Predictions were generated using five repeats of five-fold patient-grouped cross-validation, with all landmark observations from the same patient assigned to the same fold. (**A**–**C**) Patient-cluster bootstrap distributions of ROC AUC, PR AUC, and Brier score, respectively. Diamonds indicate observed grouped out-of-fold (OOF) estimates, open circles indicate bootstrap medians, thick bars indicate interquartile ranges, and thin bars represent 95% bootstrap intervals from 2000 resampling iterations. (**D**) Recalibration intercepts and calibration slopes expressed as deviations from their ideal values (intercept = 0 and slope = 1). Negative slope deviations indicate calibration slopes below 1, suggesting potential overfitting or reduced transportability. Apparent training-set performance was not used for optimism correction because only grouped OOF predictions were retained.

**Figure 10:**
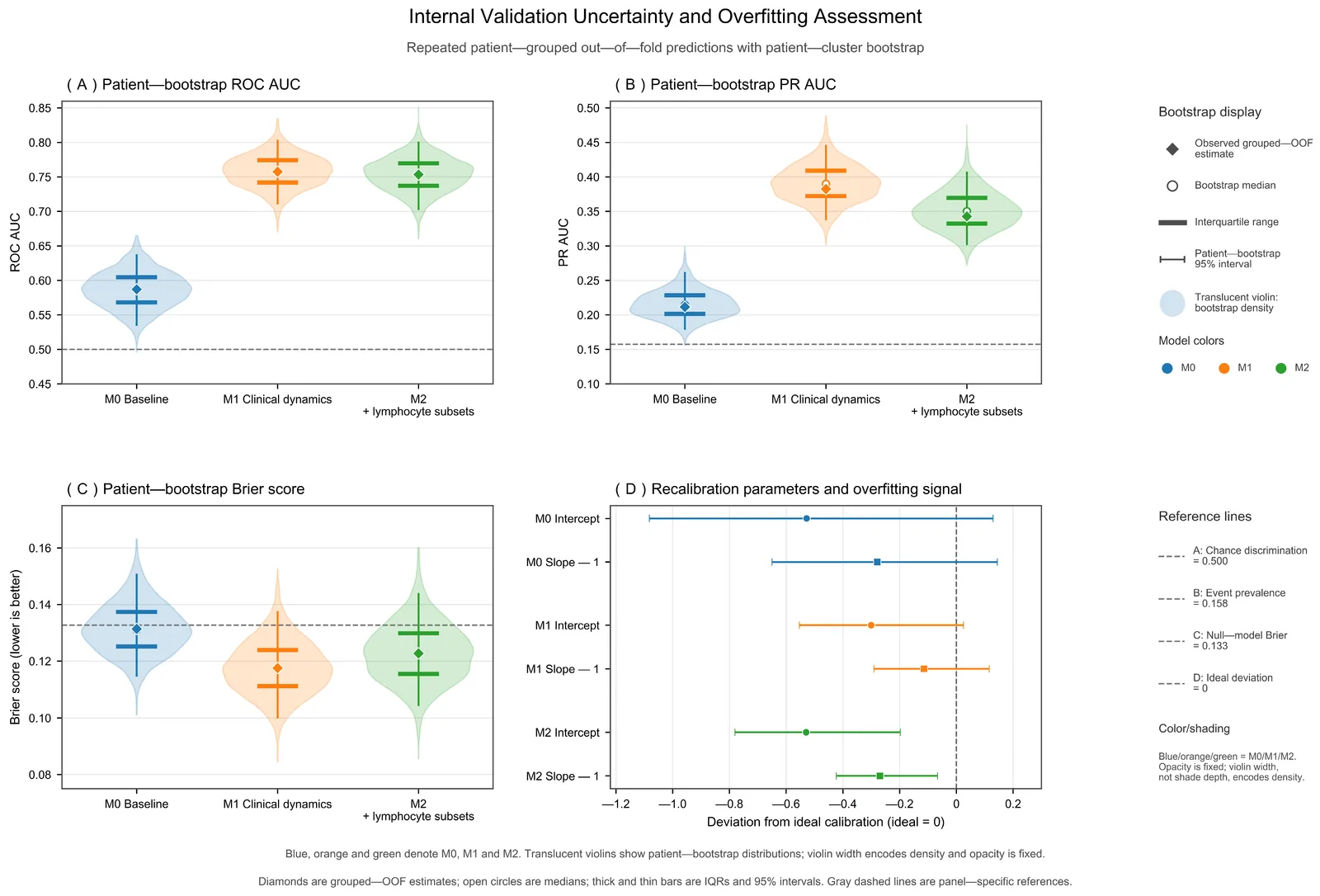
**Window-specific calibration and decision-curve analysis of dynamic landmark models.** Patient-grouped out-of-fold predictions were evaluated using patient-cluster bootstrap resampling. M0 represents the baseline model, M1 incorporates dynamic clinical features, and M2 further includes lymphocyte-subset dynamics. (**A**) Bootstrap distributions of ROC AUC for M0, M1, and M2. Diamonds indicate observed grouped out-of-fold estimates, open circles indicate bootstrap medians, thick bars represent interquartile ranges, and thin bars indicate 95% bootstrap intervals. (**B**) Bootstrap distributions of PR AUC for the three nested models. The dashed horizontal line indicates the benefit prevalence reference. (**C**) Bootstrap distributions of Brier scores, with lower values indicating better predictive performance. The dashed horizontal line represents the null-model Brier score based on event prevalence. (**D**) Recalibration parameters and potential overfitting signals, including model intercepts and slopes relative to ideal calibration (intercept = 0 and slope = 1). Error bars indicate 95% bootstrap intervals. Colors correspond to M0 (blue), M1 (orange), and M2 (green).

Longitudinal analysis of patient trajectories revealed distinct patterns of risk evolution and immune remodeling preceding PD or recurrence during PD-1 inhibitor therapy. Patients who subsequently developed PD or recurrence exhibited progressive increases in predicted risk over follow-up, whereas patients maintaining treatment benefit showed relatively stable risk profiles (Figure 9). These divergent trajectories suggest that dynamic risk estimation was able to capture gradual transitions in treatment response status before the occurrence of clinically evident disease progression, providing a temporal view of treatment failure beyond conventional endpoint-based evaluation. In parallel, longitudinal immune monitoring revealed heterogeneous patterns of immune remodeling among patients with different outcomes. Changes in lymphocyte-related features, including T-cell subsets and immune functional axes, reflected dynamic alterations in systemic immune status during treatment (Figure 10). The coordinated evolution of tumor burden, inflammatory status, and immune composition indicated that treatment response under PD-1 blockade is not a static process but involves continuous interactions between disease progression and host immune state. Together, these findings demonstrate that integrating longitudinal clinical and immune information enables a more comprehensive characterization of individual treatment trajectories and provides a potential framework for dynamic monitoring of immunotherapy response.

## 4. Discussion

This study systematically developed and internally evaluated a dynamic landmark prediction framework integrating longitudinal clinical trajectories and immune monitoring for continuous risk assessment in patients with gastric cancer receiving PD-1 inhibitor-based therapy. Unlike conventional prediction approaches that rely mainly on pretreatment characteristics and provide a fixed estimation of treatment outcome at baseline, our framework incorporated sequential information collected throughout the treatment course and enabled repeated updating of individual PD/recurrence risk according to evolving patient status. By applying a landmark analysis strategy with consecutive follow-up windows, this framework captured the time-dependent nature of immunotherapy response and allowed risk assessment to dynamically reflect changes in tumor burden, systemic inflammation, and immune status. Within this framework, three nested prediction models were established, including a baseline model based on pretreatment characteristics (M0), a dynamic clinical model incorporating longitudinal treatment-related information (M1), and an immune-integrated model further including dynamic lymphocyte subset features (M2). The results demonstrated that longitudinal clinical trajectories provided substantial predictive value beyond baseline characteristics, while the incorporation of dynamic immune features further improved individualized risk stratification. These findings indicate that integrating evolving clinical and immune information provides a more comprehensive representation of treatment response than static baseline assessment alone. Collectively, this study supports a shift from one-time risk classification toward adaptive, longitudinal monitoring strategies and provides a potential framework for individualized risk assessment and treatment surveillance during PD-1 inhibitor therapy in gastric cancer.

Current immunotherapy decision-making in gastric cancer is largely guided by pretreatment biomarkers, including PD-L1 expression, microsatellite instability/mismatch repair status, tumor mutational burden, and conventional clinicopathological characteristics [22,23,24]. These biomarkers have substantially advanced patient selection by providing important information regarding the baseline immune landscape, tumor biology, and potential sensitivity to PD-1 inhibitor therapy [25,26]. However, most currently available biomarkers are obtained before treatment initiation and therefore represent a static snapshot of disease status at a single time point. Such baseline measurements may not fully capture the dynamic interactions between tumor evolution and host immunity that continuously shape treatment response under therapeutic pressure [27]. During PD-1 blockade, clinical outcomes are influenced not only by the initial immune context but also by subsequent changes in tumor burden, systemic inflammatory responses, lymphocyte composition, immune activation, and adaptive immune remodeling [28]. Consequently, patients with similar baseline characteristics may experience substantially different treatment trajectories due to divergent biological changes occurring during therapy. Therefore, longitudinal assessment that incorporates treatment-associated changes may provide complementary information beyond conventional baseline biomarkers and better reflect the evolving state of immunotherapy response. In this context, our study extends existing biomarker-based strategies by integrating longitudinal clinical and peripheral immune information into a dynamic prediction framework, allowing individual risk assessment to be continuously updated throughout treatment and providing a more comprehensive approach for monitoring PD-1 inhibitor efficacy.

An important aspect of this study is the integration of interpretable clinical and immune trajectories into the dynamic prediction framework, allowing the model outputs to be linked with measurable biological changes during PD-1 inhibitor therapy. The incremental improvement from M0 to M1 indicates that treatment-associated clinical evolution, particularly changes in tumor burden and systemic inflammatory status, provides substantial prognostic information beyond baseline characteristics. Among these dynamic variables, tumor burden trajectories and neutrophil-to-lymphocyte ratio (NLR) changes showed prominent contributions to subsequent PD/recurrence risk, highlighting the importance of monitoring disease evolution and inflammatory responses during treatment. Furthermore, the additional predictive value gained from incorporating dynamic lymphocyte subset features in M2 suggests that peripheral immune remodeling provides complementary information for individualized risk assessment. The identified immune-related features, including T-cell compartments, innate cytotoxicity, humoral immunity, and immune balance axes, further reflect the complex and evolving host immune state during PD-1 blockade. Importantly, the interpretability analysis demonstrates that the proposed framework is not solely a statistical prediction model but connects risk estimation with clinically accessible changes in tumor burden, inflammation, and immune composition. This feature enhances model transparency and provides a biologically informed basis for dynamic monitoring and personalized management of immunotherapy response.

The clinical implications of this dynamic prediction framework extend beyond outcome prediction and provide a potential strategy for individualized monitoring and adaptive management during PD-1 inhibitor therapy. Current clinical assessment of immunotherapy response is largely based on scheduled imaging evaluations and baseline biomarker information, which may not fully reflect rapid changes in tumor behavior and host immune status during treatment [29,30]. By continuously integrating longitudinal clinical trajectories and immune alterations, the proposed framework enables dynamic reassessment of patient risk throughout the treatment course [31]. Patients exhibiting increasing predicted risk trajectories or unfavorable changes in tumor burden, inflammatory status, or immune profiles may be identified earlier and considered for intensified surveillance, additional molecular characterization, or timely therapeutic adjustment [32]. Conversely, patients with stable low-risk trajectories may continue current treatment strategies while potentially avoiding unnecessary investigations or interventions. Therefore, this framework may serve as a complementary tool to existing clinical decision-making processes by providing continuously updated risk information rather than replacing established biomarkers or treatment guidelines.

Despite these potential applications, several limitations should be acknowledged. First, this study was developed and evaluated using a single-center gastric cancer cohort with a relatively limited sample size, which may affect the generalizability of the findings and contribute to uncertainty in model estimation, particularly in later landmark windows with fewer patients and observed events. Second, although repeated patient-level cross-validation and bootstrap resampling were implemented to reduce overfitting and improve the reliability of model evaluation, independent external validation in multicenter cohorts remains necessary to confirm the robustness, reproducibility, and clinical transportability of the proposed framework. Third, as a real-world study, variations in treatment regimens, including different PD-1 inhibitor combinations, treatment lines, and subsequent therapeutic interventions, may have introduced residual clinical heterogeneity that could not be completely controlled. Fourth, although peripheral lymphocyte subset monitoring provides a practical and minimally invasive approach for longitudinal immune assessment, circulating immune profiles may not fully represent the spatial and functional complexity of the tumor immune microenvironment. Finally, the current framework focused on predicting PD/recurrence within subsequent landmark windows rather than directly modeling long-term survival outcomes. Future studies incorporating time-to-event approaches, additional molecular and imaging biomarkers, and prospective multicenter validation will be required to further optimize the framework and establish its clinical utility.

Future studies should focus on prospective multicenter validation and further refinement of dynamic risk assessment frameworks in larger and more diverse gastric cancer populations. Standardized follow-up schedules, harmonized immune monitoring protocols, and consistent definitions of clinical outcomes will be essential to evaluate the reproducibility and clinical applicability of longitudinal prediction models across different settings. Beyond peripheral lymphocyte profiling, integration of additional biological and clinical dimensions, including circulating tumor DNA dynamics, tumor genomic alterations, radiological features, pathological characteristics, and spatial immune profiling, may further improve the biological resolution and predictive accuracy of individualized risk assessment. Moreover, advances in interpretable machine learning and multimodal data integration provide new opportunities to transform longitudinal clinical information into adaptive decision-support systems that can continuously update patient risk during treatment. Ultimately, the integration of dynamic clinical trajectories, immune remodeling patterns, and multimodal biomarkers may facilitate a transition from static baseline classification toward continuously updated precision immunotherapy management, providing a foundation for more individualized treatment strategies in gastric cancer.

In conclusion, this study establishes a dynamic landmark prediction framework that shifts immunotherapy assessment from static baseline classification toward longitudinal risk evaluation during PD-1 inhibitor therapy. By integrating evolving clinical trajectories with peripheral immune remodeling, the framework enables continuous updating of PD/recurrence risk and provides a more comprehensive representation of individual treatment trajectories. The findings demonstrate that treatment-associated clinical changes and immune dynamics offer complementary predictive information beyond baseline characteristics, supporting a more adaptive approach to immunotherapy monitoring. Although external validation in larger multicenter cohorts remains required, this study provides a foundation for developing dynamic, data-driven strategies for precision immunotherapy management in gastric cancer.

## Figures and Tables

**Table 1:** Baseline characteristics of the 171 patients with gastric cancer receiving PD-1 inhibitor therapy: comparison between the benefit and non-benefit groups (47 vs. 124).

Variable	Benefit (*n* = 47)	Non-Benefit (*n* = 12)	*p* Value
Age, years	62.0 (53.5, 69.5)	60.0 (53.8, 66.0)	0.278
Baseline maximum tumor diameter, mm	46.5 (33.5, 59.3)	44.9 (35.2, 54.4)	0.623
Baseline body weight, kg	57.4 (49.7, 63.4)	61.3 (53.9, 67.7)	0.031
Baseline albumin, g/L	38.4 (36.0, 42.7)	38.6 (36.2, 41.1)	0.594
Baseline C-reactive protein, mg/L	9.3 (4.8, 12.9)	6.9 (4.5, 11.2)	0.178
Baseline hemoglobin, g/L	122.0 (107.5, 130.0)	118.0 (110.8, 129.0)	0.528
Baseline neutrophil-to-lymphocyte ratio	2.85 (2.23, 3.71)	3.05 (2.41, 3.81)	0.400
Sex, *n*/N (%)			0.886
☐Male	32/47 (68.1%)	83/124 (66.9%)	
☐Female	15/47 (31.9%)	41/124 (33.1%)	
Pathological stage, *n*/N (%)			<0.001
☐Stage II	21/47 (44.7%)	42/124 (33.9%)	
☐Stage III	0/47 (0.0%)	21/124 (16.9%)	
☐Stage IVA	1/47 (2.1%)	18/124 (14.5%)	
☐Stage IVB	25/47 (53.2%)	43/124 (34.7%)	
Histological subtype, *n*/N (%)			0.702
Adenocarcinoma	24/47 (51.1%)	63/124 (50.8%)	
Poorly differentiated adenocarcinoma	14/47 (29.8%)	43/124 (34.7%)	
Signet ring cell carcinoma	9/47 (19.1%)	18/124 (14.5%)	
Baseline ECOG performance status, *n*/N (%)			<0.001
☐0	11/47 (23.4%)	21/124 (16.9%)	
☐1	18/47 (38.3%)	85/124 (68.5%)	
☐2	17/47 (36.2%)	18/124 (14.5%)	
☐3	1/47 (2.1%)	0/124 (0.0%)	
Previous surgery, *n*/N (%)			0.438
☐Yes	20/47 (42.6%)	61/124 (49.2%)	
☐No	27/47 (57.4%)	63/124 (50.8%)	

Note: Continuous variables are presented as median (interquartile range) and were compared using the two-sided Mann–Whitney U test. Categorical variables were compared using the Pearson chi-square test; Fisher’s exact test was applied when the expected frequency in a 2 × 2 contingency table was <5.

**Table 2:** Baseline characteristics of the 171-patient gastric cancer PD-1 cohort: comparison between the recurrence group and the non-recurrence group (48 vs. 123).

Variable	Benefit (*n* = 47)	Non-Benefit (*n* = 12)	*p* Value
Age, years	55.5 (53.8, 61.0)	62.0 (53.5, 68.0)	0.007
Baseline maximum tumor diameter, mm	42.2 (31.5, 48.2)	47.5 (35.5, 59.7)	0.007
Baseline body weight, kg	64.7 (57.6, 68.5)	58.7 (51.5, 65.5)	0.022
Baseline albumin, g/L	40.1 (37.4, 42.2)	38.3 (36.0, 41.0)	0.027
Baseline C-reactive protein, mg/L	5.5 (3.9, 9.9)	8.4 (5.0, 12.5)	0.022
Baseline hemoglobin, g/L	120.5 (114.8, 133.2)	118.0 (109.0, 128.5)	0.057
Baseline neutrophil-to-lymphocyte ratio	2.94 (2.36, 3.32)	3.11 (2.35, 3.88)	0.106
Sex, *n*/N (%)			0.794
☐Male	33/48 (68.8%)	82/123 (66.7%)	
☐Female	15/48 (31.2%)	41/123 (33.3%)	
Pathological stage, *n*/N (%)			0.002
☐Stage II	27/48 (56.2%)	36/123 (29.3%)	
☐Stage III	7/48 (14.6%)	14/123 (11.4%)	
☐Stage IVA	5/48 (10.4%)	14/123 (11.4%)	
☐Stage IVB	9/48 (18.8%)	59/123 (48.0%)	
Histological subtype, *n*/N (%)			0.242
Adenocarcinoma	26/48 (54.2%)	61/123 (49.6%)	
Poorly differentiated adenocarcinoma	18/48 (37.5%)	39/123 (31.7%)	
Signet ring cell carcinoma	4/48 (8.3%)	23/123 (18.7%)	
Baseline ECOG performance status, *n*/N (%)			0.255
☐0	13/48 (27.1%)	19/123 (15.4%)	
☐1	28/48 (58.3%)	75/123 (61.0%)	
☐2	7/48 (14.6%)	28/123 (22.8%)	
☐3	0/48 (0.0%)	1/123 (0.8%)	
Previous surgery, *n*/N (%)			0.013
☐Yes	30/48 (62.5%)	51/123 (41.5%)	
☐No	18/48 (37.5%)	72/123 (58.5%)	

Note: Continuous variables are presented as median (interquartile range [IQR]) and were compared using the two-sided Mann–Whitney U test. Categorical variables were compared using Pearson’s chi-square test; Fisher’s exact test was applied when the expected frequency in a 2 × 2 contingency table was <5. The “non-recurrence group” included patients with final outcomes classified as PR, SD, or PD whose labels were not annotated as “Recurrence”.

**Table 3:** Landmark cohort composition, longitudinal data coverage, and quality control for v9 (initial cohort, *n* = 171). (**A**) Landmark sample composition and longitudinal data coverage. (**B**) Quality control and sensitivity considerations for the landmark analysis.

(A)						
Window	Date Range	Risk Set (*n*)	Proportion of Initial Cohort	PD/Recurrence Events Within Window (*n*)	Patients with Follow-Up Data, *n*/N (%)	Patients with Lymphocyte Subset Data, *n*/N (%)
W1	D1–28	171	100.0%	12	171/171 (100.0%)	171/171 (100.0%)
W2	D29–56	159	93.0%	21	159/159 (100.0%)	159/159 (100.0%)
W3	D57–84	138	80.7%	18	138/138 (100.0%)	138/138 (100.0%)
W4	D85–112	120	70.2%	18	120/120 (100.0%)	120/120 (100.0%)
W5	D113–140	102	59.6%	22	102/102 (100.0%)	102/102 (100.0%)
W6	D141–168	80	46.8%	17	80/80 (100.0%)	80/80 (100.0%)
W7	D169–196	63	36.8%	14	63/63 (100.0%)	63/63 (100.0%)
W8	D197–224	49	28.7%	2	49/49 (100.0%)	49/49 (100.0%)
**(B)**						
**Metric**			**Value**	**Proportion/Range**	**Description**	
Initial PD-1 cohort			171 patients	100.0%	All patients had gastric cancer	
Entered at least one subsequent landmark risk set			159 patients	93.0%	Remained free of PD/recurrence at the end of W1	
Landmark patient-window records			711 records	Not applicable	Pooled across W1–W7	
First PD/recurrence events in the subsequent window			112 events	15.8%	Calculated based on patient-window records	
Evaluable landmark windows			7 windows	W1–W7	W8 lacked W9 outcomes and could not be used to evaluate subsequent-window performance	
Patient-level internal validation			5-fold × 5 repetitions	Not applicable	All windows from the same patient were consistently assigned to the same data partition	
W7 risk set and event count			49 patients/2 events	4.1% event rate	Low event count; interpreted for exploratory purposes only	

Note: W1 represents days 1–28 (D1–28), W2 represents days 29–56 (D29–56), and subsequent windows were defined sequentially through W8 (D197–224). The risk set included patients who remained free of PD or recurrence at the start of each landmark window.

**Table 4:** Predictive performance, paired model comparisons, and window-specific landmark analyses for v9.

Analysis Category	Model/Comparison	Analysis Cohort/Time Window	*n*	Events (*n*)	Event Rate	ROC AUC or Δ (95% CI)	PR AUC or Δ (95% CI)	Brier Score or Δ (95% CI)	Statistical Method/Description
Final clinical benefit outcome models	M0 baseline model	Full cohort (*n* = 171)	171	47	27.5%	0.559 (0.429–0.690)	0.625 (0.507–0.740)	0.195 (0.182–0.208)	Patient-level bootstrap 95% CI
	M1 clinical dynamic model	Full cohort (*n* = 171)	171	47	27.5%	0.737 (0.613–0.852)	0.763 (0.661–0.866)	0.192 (0.188–0.196)	Patient-level bootstrap 95% CI
	M2 clinical dynamic + lymphocyte subset model	Full cohort (*n* = 171)	171	47	27.5%	0.786 (0.681–0.883)	0.791 (0.692–0.884)	0.188 (0.184–0.192)	Patient-level bootstrap 95% CI
Paired comparisons of nested models	M1−M0	Paired predictions in 171 patients	171	47	27.5%	+0.178 (0.025–0.326); *p* = 0.025	+0.138 (0.005–0.270); *p* = 0.046	−0.003 (−0.015–0.010); *p* = 0.645	Δ metrics; paired DeLong test for ROC, patient-level bootstrap for other metrics
	M2−M1	Paired predictions in 171 patients	171	47	27.5%	+0.049 (0.008–0.101); *p* = 0.040	+0.028 (−0.013–0.075); *p* = 0.209	−0.004 (−0.005–−0.003); *p* < 0.001	Δ metrics; paired DeLong test for ROC, patient-level bootstrap for other metrics
	M2−M0	Paired predictions in 171 patients	171	47	27.5%	+0.227 (0.091–0.363); *p* = 0.002	+0.166 (0.037–0.291); *p* = 0.011	−0.007 (−0.019–0.005); *p* = 0.275	Δ metrics; paired DeLong test for ROC, patient-level bootstrap for other metrics
Subsequent-window Landmark models	M0 baseline model	Pooled W1–W7; 159 patients/711 records	711	112	15.8%	0.587 (0.531–0.642)	0.212 (0.175–0.255)	0.131 (0.113–0.148)	500-round patient-cluster bootstrap
	M1 clinical longitudinal model	Pooled W1–W7; 159 patients/711 records	711	112	15.8%	0.758 (0.709–0.799)	0.383 (0.337–0.445)	0.118 (0.099–0.138)	500-round patient-cluster bootstrap
	M2 clinical longitudinal + lymphocyte subset model	Pooled W1–W7; 159 patients/711 records	711	112	15.8%	0.753 (0.705–0.796)	0.343 (0.303–0.415)	0.123 (0.104–0.144)	500-round patient-cluster bootstrap
Window-specific landmark models	M0 baseline model	W1 → W2 prediction	159	21	13.2%	0.551 (0.390–0.714)	0.185 (0.111–0.333)	0.118 (0.086–0.152)	500-round patient-cluster bootstrap
	M1 clinical longitudinal model	W1 → W2 prediction	159	21	13.2%	0.539 (0.428–0.665)	0.160 (0.094–0.302)	0.122 (0.084–0.162)	500-round patient-cluster bootstrap
	M2 clinical longitudinal + lymphocyte subset model	W1 → W2 prediction	159	21	13.2%	0.541 (0.432–0.655)	0.152 (0.094–0.285)	0.125 (0.087–0.167)	500-round patient-cluster bootstrap
	M0 baseline model	W2 → W3 prediction	138	18	13.0%	0.567 (0.428–0.704)	0.180 (0.101–0.355)	0.117 (0.083–0.153)	500-round patient-cluster bootstrap
	M1 clinical longitudinal model	W2 → W3 prediction	138	18	13.0%	0.606 (0.479–0.720)	0.161 (0.098–0.273)	0.127 (0.088–0.169)	500-round patient-cluster bootstrap
	M2 clinical longitudinal + lymphocyte subset model	W2 → W3 prediction	138	18	13.0%	0.619 (0.475–0.740)	0.172 (0.102–0.288)	0.135 (0.099–0.174)	500-round patient-cluster bootstrap
	M0 baseline model	W3 → W4 prediction	120	18	15.0%	0.603 (0.460–0.742)	0.203 (0.115–0.371)	0.129 (0.087–0.171)	500-round patient-cluster bootstrap
	M1 clinical longitudinal model	W3 → W4 prediction	120	18	15.0%	0.682 (0.582–0.785)	0.236 (0.138–0.419)	0.132 (0.098–0.171)	500-round patient-cluster bootstrap
	M2 clinical longitudinal + lymphocyte subset model	W3 → W4 prediction	120	18	15.0%	0.708 (0.595–0.808)	0.261 (0.150–0.469)	0.131 (0.099–0.168)	500-round patient-cluster bootstrap
	M0 baseline model	W4 → W5 prediction	102	22	21.6%	0.468 (0.339–0.599)	0.221 (0.147–0.367)	0.176 (0.127–0.231)	500-round patient-cluster bootstrap
	M1 clinical longitudinal model	W4 → W5 prediction	102	22	21.6%	0.720 (0.598–0.824)	0.369 (0.245–0.573)	0.158 (0.120–0.206)	500-round patient-cluster bootstrap
	M2 clinical longitudinal + lymphocyte subset model	W4 → W5 prediction	102	22	21.6%	0.688 (0.568–0.785)	0.310 (0.214–0.475)	0.172 (0.133–0.220)	500-round patient-cluster bootstrap
	M0 baseline model	W5 → W6 prediction	80	17	21.2%	0.658 (0.515–0.796)	0.402 (0.205–0.640)	0.159 (0.105–0.219)	500-round patient-cluster bootstrap
	M1 clinical longitudinal model	W5 → W6 prediction	80	17	21.2%	0.899 (0.822–0.958)	0.649 (0.438–0.864)	0.108 (0.073–0.150)	500-round patient-cluster bootstrap
	M2 clinical longitudinal + lymphocyte subset model	W5 → W6 prediction	80	17	21.2%	0.868 (0.775–0.938)	0.570 (0.377–0.801)	0.122 (0.081–0.171)	500-round patient-cluster bootstrap
	M0 baseline model	W6 → W7 prediction	63	14	22.2%	0.752 (0.574–0.910)	0.597 (0.362–0.836)	0.158 (0.099–0.227)	500-round patient-cluster bootstrap
	M1 clinical longitudinal model	W6 → W7 prediction	63	14	22.2%	0.968 (0.927–0.999)	0.879 (0.693–0.996)	0.082 (0.050–0.114)	500-round patient-cluster bootstrap
	M2 clinical longitudinal + lymphocyte subset model	W6 → W7 prediction	63	14	22.2%	0.981 (0.948–1.000)	0.937 (0.806–1.000)	0.069 (0.041–0.096)	500-round patient-cluster bootstrap
	M0 baseline model	W7 → W8 prediction	49	2	4.1%	0.521 (0.043–1.000)	0.189 (0.022–1.000)	0.051 (0.022–0.099)	Only two events were available, leading to highly unstable results.
	M1 clinical longitudinal model	W7 → W8 prediction	49	2	4.1%	1.000 (1.000–1.000)	1.000 (1.000–1.000)	0.021 (0.009–0.035)	Only two events were available, leading to highly unstable results.
	M2 clinical longitudinal + lymphocyte subset model	W7 → W8 prediction	49	2	4.1%	0.979 (0.935–1.000)	0.583 (0.333–1.000)	0.028 (0.010–0.054)	Only two events were available, leading to highly unstable results.

Note: The final clinical benefit outcome was defined as PR/SD. The subsequent-window landmark outcome was defined as the first occurrence of PD or recurrence during W(k + 1). A negative ΔBrier indicates improvement by the candidate model. Confidence intervals for the window-specific analyses were obtained using 500-repeat patient-cluster bootstrap.

**Table 5:** Dynamic lymphocyte subset features and immune functional axis variables for v9.

Variable Name	Functional Axis	Original Interpretation	Directional analysis of v9
current_tumor_change_pct	Tumor burden change axis	Tumor size change from baseline in current window (%)	β = +0.732, OR = 2.080; Risk ↑; Rank 1
slope_tumor_change	Tumor burden change axis	Tumor size change slope up to current window	β = −0.321, OR = 0.726; Risk ↓; Rank 12
current_nlr	Systemic inflammation axis	NLR in current window	β = +0.410, OR = 1.507; Risk ↑; Rank 7
slope_nlr	Systemic inflammation axis	NLR change slope up to current window	β = +0.166, OR = 1.180; Risk ↑; Rank 19
current_lymph_abs	Lymphocyte reserve axis	Absolute lymphocyte count in current window	β = +0.052, OR = 1.053; Risk ↑; Rank 33
slope_lymph_abs	Lymphocyte reserve axis	Absolute lymphocyte count change slope	β = −0.037, OR = 0.963; Risk ↓; Rank 36
current_cd3_t_pct	Total T-cell axis	CD3^+^ T-cell proportion in current window	β = −0.373, OR = 0.688; Risk ↓; Rank 9
slope_cd3_t_pct	Total T-cell axis	CD3^+^ T-cell proportion change slope	β = +0.033, OR = 1.034; Risk ↑; Rank 37
current_cd4_t_pct	CD4^+^ T-cell axis	CD4^+^ T-cell proportion in current window	β = +0.130, OR = 1.139; Risk ↑; Rank 22
slope_cd4_t_pct	CD4^+^ T-cell axis	CD4^+^ T-cell proportion change slope	β = −0.038, OR = 0.963; Risk ↓; Rank 35
current_cd8_t_pct	CD8^+^ T-cell axis	CD8^+^ T-cell proportion in current window	β = −0.276, OR = 0.759; Risk ↓; Rank 14
slope_cd8_t_pct	CD8^+^ T-cell axis	CD8^+^ T-cell proportion change slope	β = +0.008, OR = 1.008; Risk ↑; Rank 43
current_cd4_cd8_ratio	Immune balance axis	CD4/CD8 ratio in current window	β = −0.148, OR = 0.863; Risk ↓; Rank 21
slope_cd4_cd8_ratio	Immune balance axis	CD4/CD8 ratio change slope	β = +0.209, OR = 1.233; Risk ↑; Rank 17
current_nk_pct	Innate cytotoxicity axis	NK-cell proportion in current window	β = +0.039, OR = 1.040; Risk ↑; Rank 34
slope_nk_pct	Innate cytotoxicity axis	NK-cell proportion change slope	β = −0.030, OR = 0.971; Risk ↓; Rank 39
current_b_cell_pct	Humoral immunity axis	CD19^+^ B-cell proportion in current window	β = +0.214, OR = 1.239; Risk ↑; Rank 16
slope_b_cell_pct	Humoral immunity axis	CD19^+^ B-cell proportion change slope	β = −0.154, OR = 0.857; Risk ↓; Rank 20

Note: β coefficients and ORs were derived from the standardized coefficients of the pooled landmark M2 model and were used only for directional interpretation in the synthetic dataset. Risk ↑/↓ indicates the direction of association between increased variable values and the risk of PD/recurrence in the subsequent window.

## Data Availability

The datasets generated and analyzed during the current study are available from the corresponding author upon reasonable request. Because the dataset contains potentially identifiable patient-level clinical information and was collected under institutional ethical approval, the raw data cannot be publicly deposited. Requests for data access will be evaluated according to institutional policies and applicable ethical regulations.

## Abbreviations

AI, artificial intelligence; AP, average precision; AUC, area under the receiver operating characteristic curve; CI, confidence interval; CR, complete response; CT, computed tomography; DFS, disease-free survival; dMMR, deficient mismatch repair; ECOG, Eastern Cooperative Oncology Group; GC, gastric cancer; HER2, human epidermal growth factor receptor 2; ICI, immune checkpoint inhibitor; IQR, interquartile range; MMR, mismatch repair; MRI, magnetic resonance imaging; MSI-H, microsatellite instability-high; OS, overall survival; PD-1, programmed cell death protein 1; PD-L1, programmed death-ligand 1; PFS, progression-free survival; PR, partial response; RECIST, Response Evaluation Criteria in Solid Tumors; SD, stable disease; TIME, tumor immune microenvironment; TNM, tumor-node-metastasis.
